# A prospective cohort study on postoperative radiotherapy with TomoDirect using simultaneous integrated boost technique in early breast cancer

**DOI:** 10.1186/s13014-014-0244-0

**Published:** 2014-11-19

**Authors:** Hyo Chun Lee, Sung Hwan Kim, Young Jin Suh, Mi Joo Chung, Dae Gyu Kang, Hyun Joo Choi, Jong Hoon Lee

**Affiliations:** Department of Radiation Oncology, St. Vincent’s Hospital, College of Medicine, The Catholic University of Korea, Seoul, Republic of Korea, 442-723, 93-6, Ji-dong, Paldal-gu, Suwon, Kyeonggi-do Republic of Korea; Department of Surgery, St. Vincent’s Hospital, The Catholic University of Korea, Seoul, Republic of Korea; Department of Hospital Pathology, St. Vincent’s Hospital, The Catholic University of Korea, Seoul, Republic of Korea

**Keywords:** Breast cancer, Radiotherapy, TomoTherapy, Toxicity

## Abstract

**Purpose:**

To evaluate the technical feasibility and toxicity of TomoDirect in breast cancer patients who received radiotherapy after breast-conserving surgery.

**Methods:**

155 consecutive patients with breast carcinoma in situ or T1-2 breast cancer with negative lymph node received breast irradiation with TomoDirect using simultaneous integrated boost technique in the prospective cohort study. A radiation dose of 50.4 Gy and 57.4 Gy in 28 fractions was prescribed to the ipsilateral breast and tumor bed, respectively. Dosimetric parameters of target and organ at risk and acute complication were assessed prospectively.

**Results:**

The mean dose for the tumor bed is 58.90 Gy. The mean values of V_54.53Gy (95% of the prescribed dose)_, V_63.14Gy (110% of the prescribed dose)_, and V_66.01Gy (115% of the prescribed dose)_ were 99.97%, 1.26%, and 0%, respectively. The mean value of radiation conformality index was 1.01. The mean value of radical dose homogeneity index was 0.89. The average dose irradiated to the ipsilateral lung, heart, and contralateral breast was 4.72 Gy, 1.09 Gy, and 0.19 Gy, respectively. The most common toxicity was dermatitis. During breast irradiation, grade 2 and 3 dermatitis occurred in 41 (26.5%) and 6 (3.9%) of the 155 patients, respectively. Two patients had arm lymphedema during breast irradiation. Two patients had grade 2 pneumonitis 1 month after breast irradiation.

**Conclusions:**

Radiotherapy using TomoDirect in early breast cancer patients showed acceptable toxicities and optimal results in terms of target coverage and organ at risk sparing.

## Introduction

Breast cancer is the frequently diagnosed cancer in South Korea and it accounts for about 15% of all female malignancies [1]. The annual incidence of breast cancer has been gradually rising primarily due to increased utilization of screening mammography. Breast-conserving surgery and postoperative radiotherapy are basic treatment modalities that have been widely used to manage early breast cancer on the basis of the results of randomized prospective trials [2]. The long-term survival rate among women who undergo breast-conserving surgery followed by breast irradiation is the same as that among women who undergo radical mastectomy.

Conventional breast radiotherapy consists of 45 to 50 Gy of whole breast irradiation performed using two tangential megavoltage photon beams and 10 to 16 Gy of boost irradiation delivered to the tumor bed with electron beams. Recently, techniques to improve the accuracy of radiation delivery to the target have advanced. Helical TomoTherapy (Accuary, Sunnyvale, CA), which can involve image-guided radiation therapy (IGRT) using a megavoltage computed tomography (CT) scan just prior to radiation treatment, is one specific example of these advancements. TomoTherapy can also yield intensity-modulated radiation therapy (IMRT) that allows for highly conformal distributions of radiation dose to the target and minimizes the irradiation to adjacent dose-limiting organs. IMRT using TomoTherapy enables the simultaneous delivery of different dose prescriptions to different target volumes in the same treatment fraction. This technique is called simultaneous integrated boost (SIB). This modality is highly effective and safe in the treatment of head and neck cancer by reducing the overall treatment time and increasing the fraction size to boost volumes [3].

In breast cancer patients, helical TomoTherapy is not a suitable option since the gantry continuously rotates around the patient, and this technique can deliver low-dose radiation to lungs that is associated with an occurrence of radiation pneumonitis [4]. To avoid this inefficiency of beam usage, a TomoDirect option using static gantry positions combined with simultaneous couch translation and dynamic collimator modulation has been developed. In a pilot study, TomoDirect seemed particularly well suited for postoperative irradiation in breast cancer patients [5]. TomoDirect achieved an optimal target volume coverage and coincident adequate normal tissue sparing in a dosimetric study [6].

Clinical studies of TomoDirect in breast cancer patients are scarce and have been assessed only in small and retrospective series [5,6]. Thus, we undertook a prospective study on the technical feasibility and toxicity of TomoDirect in breast cancer patients who received postoperative radiotherapy.

## Methods

### Patients

166 consecutive patients with breast carcinoma in situ (pTisN0) or early breast cancer with negative axillary node (pT1-2 N0) who underwent breast-conserving surgery between January 2012 and February 2013 were included in this prospective study. They received postoperative radiotherapy with TomoDirect. The procedures followed were in accordance with the ethical standards of the responsible committee on human experimentation in the Catholic University of Korea. Exclusion criteria were metastatic breast cancer, previous radiation history of chest wall due to the thoracic malignancy, and involved resection margin.

### Simulation and target volume contouring

During simulation, patients were immobilized in the supine position using a foam cushion, which covered the upper body and both arms. The patient’s arms were raised above the head. A contrast-enhanced CT was scanned for the treatment plan. CT imaging ranging from cervical to lumbar vertebral body was obtained at 3-mm thicknesses and was imported to the Pinnacle^3^ treatment planning system (Philips Radiation Oncology Systems, Fitchburg, WI). The following structures were contoured: clinical target volume (CTV), planning target volume (PTV), and organ at risk (OAR). CTV1 is glandular tissue of the breast. CTV2 is tumor bed cavity consisting of surgical clips, postoperative change, and seroma. CTV was identified and contoured on the axial CT images. CTV was consistently expanded by 6-mm radial and craniocaudal margin to create the PTV with a constraint of reverse expansion of 4 mm to the skin surface to avoid the potential skin toxicity [7]. Heart and lung were excluded from the PTV when needed. The PTV provided a margin around the CTV to compensate for the variability of setup during breast irradiation and motion of breast or chest with breathing. The OARs such as lung, heart, and contralateral breast were contoured. For the IMRT plan with TomoDirect, the raw dosimetric data set of each patient was transferred from the Pinnacle^3^ treatment planning system to the TomoTherapy Hi-Art version 4.0 planning system (Accuray, Sunnyvale, CA).

### Dose prescription and constraint and planning

**Figure 1:**
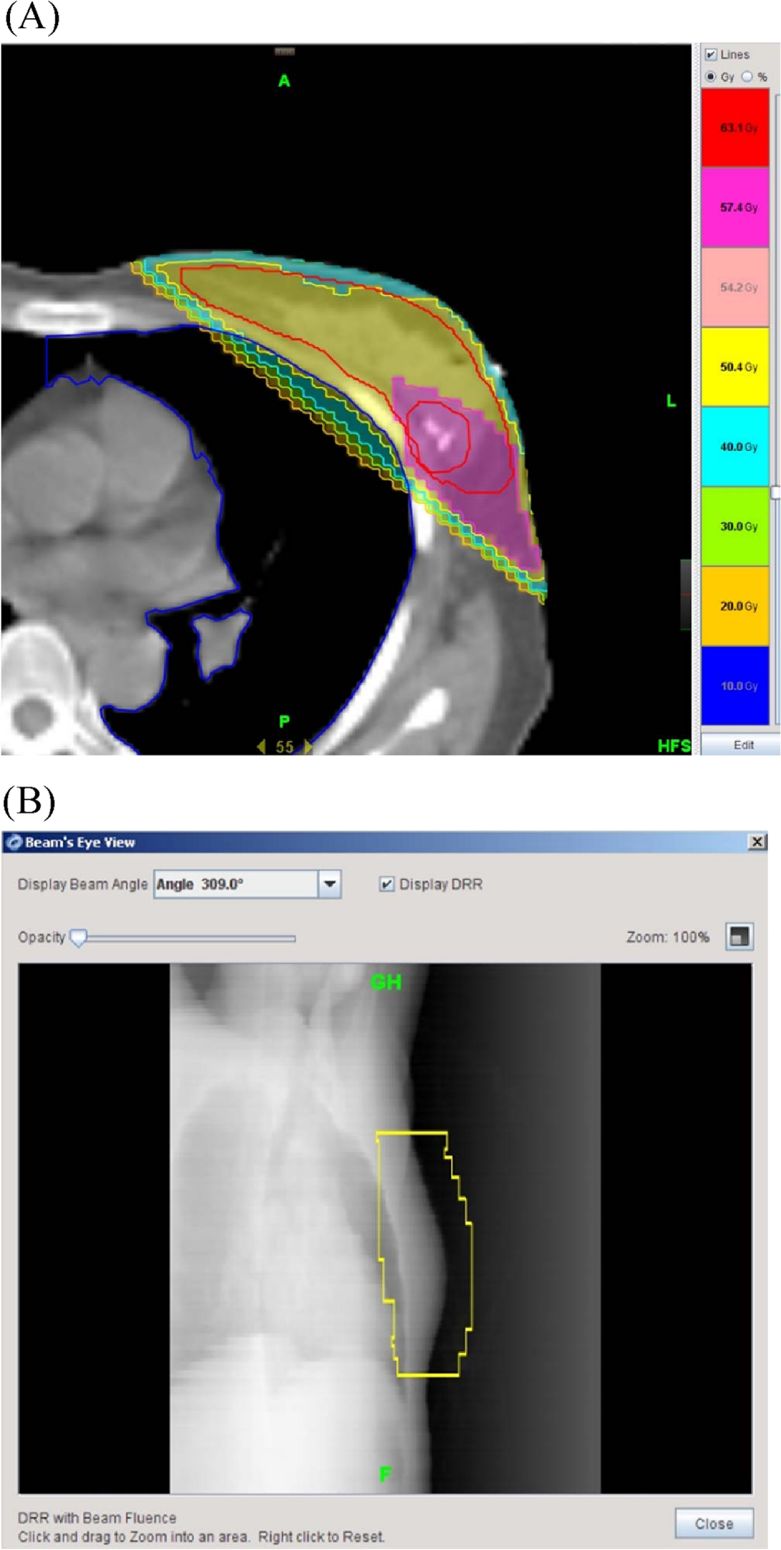
**A breast cancer patient received breast irradiation with TomoDirect using simultaneous integrated boost technique after breast-conserving surgery. A**. TomoDirect could deliver radiation dose of 50.4 Gy (outline in yellow) to the breast and 57.4 Gy (outlined in pink) in 28 fractions to the tumor bed at the same time. **B**. Beam’s eye view for the TomoDirect planning was digitally reconstructed using beam-fluence data.

TomoDirect plan for a patient with early breast cancer is shown in Figure 1. All patients were treated with the SIB technique of TomoDirect. A radiation dose of 50.4 Gy in 28 fractions was prescribed to the PTV1, and a radiation dose of 57.4 Gy in 28 fractions was prescribed to the PTV2. Dose constraints for the PTV were: (1) ≥98% of the PTV receives ≥95% of the prescribed dose and (2) ≤5% of the PTV receives ≥110% of the prescribed dose. The dose constraints for ipsilateral lung specified that the lung should receive the mean dose of ≤10 Gy, 20% of the lung was kept under 20 Gy, and 10% of the lung was kept under 30 Gy. The dose constraints for heart specified that 10% of the heart was kept under 10 Gy and 5% of the heart was kept under 20 Gy. Every effort was made to decrease the irradiated volume of organs at risk such as contralateral breast and lung as low as possible. Dosimetric parameters to analyze target coverage and dose distribution in the PTV were: (1) mean dose, (2) V_nGy_, percentage of the volume receiving radiation ≥ n Gy, (3) D_min_, minimum dose irradiated to the PTV, (4) D_max_, maximum dose irradiated to the PTV, (5) Radiation conformality index (RCI), PTV/V_95%_ (volume enclosed by the 95% of isodose line), and (6) Radical dose homogeneity index (rDHI), D_min_/D_max_ in the PTV. The irradiation to the OARs, such as lung, heart, and contralateral breast, were evaluated using values including mean dose and V_nGy_. Two tangential beams with a jaw width of 2.5 cm were used. The pitch value was set to the default value one-tenth that of the field width (0.25 cm/projection for the 2.5 cm beam). Beam angles were selected to minimize dose to OARs and avoid irradiation to the contralateral breast. To ensure that the prescribed dose was delivered to the target, three leaves of the multi-leaf collimator were opened on the anterior edge of the beams, which compensated for the possible target movement by breathing during irradiation.

### Endpoint and toxicity assessment

The primary endpoint of the present study was to estimate the dosimetric parameters of target coverage, homogeneity, and conformality and dose to organ at risk in breast cancer patients who received postoperative radiotherapy with TomoDirect. We also assess the fulfillment of dose constraints for PTV and OAR. Dose violation within PTV and OAR of 5.1% to 10% was considered a minor one and greater than 10% was scored as a major one [8]. The secondary endpoint was to assess the acute complication. During the course of radiotherapy, patients were evaluated weekly to assess acute toxicities. Patients were also followed 1, 2 and 3 months after completion of radiotherapy to assess radiation toxicities. Evaluation consisted of clinical examination, complete blood counts, and chest radiography at each visit. Adverse effects of radiotherapy were assessed using the Common Terminology Criteria for Adverse Events (version 3.0). Incidence of toxicity grade ≥2 was recorded.

### Consent

Written informed consent was obtained from the patient for publication of this report and any accompanying images.

## Results

166 consecutive patients with primary breast cancer or carcinoma in situ who had received postoperative radiotherapy using TomoDirect at our institution were enrolled. Of the 166 patients, eleven patients were excluded from the study; two patients had a metastatic disease and they received chemotherapy after breast irradiation; nine patients had involved resection margins and they received radiation dose of >57.4 Gy. Thus, the remaining 155 patients were included for the final analysis in the present study. The patient characteristics are shown in Table 1. The median age of the patients was 51 years (range, 20–83 years). They were all women. 52 (33.6%) patients had carcinoma in situ lesions and 103 (66.4%) patients had pT1-2 N0 lesions. Fifty-three (34.2%) patients received neoadjuvant (n = 12) or adjuvant chemotherapy (n = 53) before breast irradiation. All patients received the prescribed doses of radiotherapy. There were no unplanned interruptions in radiotherapy due to the treatment toxicity.

**Table 1:** **Patient and treatment characteristics (** ***n*** **= 155)**

**Characteristic**	**No.**	**%**
Age (year)		
21-40	19	12.3
41-50	54	34.9
51-60	46	29.6
61-83	36	23.2
Site		
Left	82	52.9
Right	73	47.1
T classification		
pTis	52	33.5
pT1	84	54.1
pT2	19	12.4
N classification		
Negative	155	100
Positive	0	0
Axillary lymph node dissection or sentinel lymph node biopsy	103	66.4
Neoadjuvant or adjuvant chemotherapy	53	34.2

### Dose distribution of the planning target volume

The median of the PTV contoured in the 115 patients was 345 cc (range, 135–845 cc) for PTV1 and 16 cc (range, 8–67 cc) for PTV2. The TomoDirect plan met the prescription requirements for the PTV in 154 (99.3%) of 155 patients. In one patient with a huge breast (breast volume, 845 cc), ≥98% of the PTV received 88% of the prescribed dose so that ≤5% of the PTV can receive ≥110% of the prescribed dose. The TomoDirect plan met the prescription requirements for the ipsilateral lung and heart in all cases. The median beam-on time for TomoDirect was 175 seconds (range, 125–211 seconds). Dosimetric parameters for the PTV in the TomoDirect plan are listed in Table 2. We used SIB technique using TomoDirect in breast cancer. Since PTV 1 and PTV2 are not separate volumes, and PTV2 of tumor bed exists within the PTV1 of normal breast. It is impossible to keep the target dose guideline for the PTV1 which contains the PTV2 due to the radiation build-up region near the PTV2. Thus, we did not substrate the volume of PTV1 from PTV2 and analyzed the dosimetric parameters of PTV1, and we suggest only dosimetric parameters of PTV2 [4]. The mean dose for the PTV2 was 58.90 Gy. The mean of V_54.5Gy (95% of the prescribed dose)_ and V_63.1Gy (110% of the prescribed dose)_ was 99.97% and 1.26%, respectively. The average RCI value was 1.01 and V_66.01Gy (115% of the prescribed dose)_ value was zero.

**Table 2:** **Dosimetric parameters for the planning target volume**

**Parameter**	**Mean value**
Mean dose (Gy)	58.90 ± 0.43
V_54.5Gy_ (%)	99.97 ± 0.01
V_60.2Gy_ (%)	9.19 ± 2.17
V_63.1Gy_ (%)	1.26 ± 0.91
V_66.0Gy_ (%)	0
D_min_ (Gy)	54.25 ± 1.77
D_max_ (Gy)	60.87 ± 0.91
RCI	1.01 ± 0.01
rDHI	0.89 ± 0.03

*RCI* radiation conformality index.

*rDHI* radical dose homogeneity index.

*D*_*min*_ minimum dose irradiated to the planning target volume.

*D*_*max*_ maximum dose irradiated to the planning target volume.

*V*_*nGy*_ percentage of the volume receiving radiation ≥ n Gy.

Average D_min_ and D_max_ value was 54.25 Gy and 60.87 Gy, respectively. Average rDHI value was 0.89.

### Avoidance of organs at risk

Table 3 shows the irradiated dose to the organs at risk, which included the ipsilateral lung, heart, and contralateral breast. The mean dose for the ipsilateral lung, heart, and contralateral breast was 4.72 Gy, 1.09 Gy, and 0.19 Gy, respectively. We investigated the dosimetric parameters of the ipsilateral lung, which is the most critical organ in breast cancer patients who receive postoperative breast irradiation. The mean V_50Gy_ and V_40Gy_ values of ipsilateral lung for high-dose irradiation with TomoDirect were <5%, and the mean V_20Gy_ and V_10Gy_ values for low-dose irradiation were 7.23% and 9.42%, respectively. The mean V_10-50Gy_ values for the heart and contralateral breast were <1%.

**Table 3:** **Dosimetric parameters for the organ at risk**

**Organ at risk**	**Mean value**
Ipsilateral lung	
Mean dose (Gy)	4.72 ± 5.16
V_50Gy_ (%)	0.93 ± 0.88
V_40Gy_ (%)	3.57 ± 1.93
V_30Gy_ (%)	5.33 ± 2.32
V_20Gy_ (%)	7.23 ± 2.95
V_10Gy_ (%)	9.42 ± 3.56
Heart	
Mean dose (Gy)	1.09 ± 0.85
V_50Gy_ (%)	0.11 ± 0.36
V_40Gy_ (%)	0.31 ± 0.71
V_30Gy_ (%)	0.54 ± 1.05
V_20Gy_ (%)	0.87 ± 1.50
V_10Gy_ (%)	1.21 ± 1.84
Contralateral breast	
Mean dose (Gy)	0.19 ± 0.11
V_50Gy_ (%)	0.04 ± 0.07
V_40Gy_ (%)	0.08 ± 0.13
V_30Gy_ (%)	0.17 ± 0.26
V_20Gy_ (%)	0.25 ± 0.31
V_10Gy_ (%)	0.42 ± 0.54

*V*_*nGy*_ percentage of the volume receiving radiation ≥ n Gy.

### Early treatment toxicity

Grade 2 or higher acute toxicities observed during treatment and after irradiation are listed in Table 4. No grade 4 toxicity occurred in this study. No grade 2 or higher hematologic adverse effect developed. The most common non-hematologic toxicity was dermatitis. During breast irradiation, grade 2 and 3 dermatitis occurred in forty one (26.5%) and six (3.9%) of the 155 patients, respectively. One patient who committed a minor violation of target dose had grade 2 dermatitis without other acute toxicities. There was no significant difference between six patients (3.9%) with grade 3 dermatitis and others in term of mean PTV dose, RCI, and rDHI. All skin problems were recovered with conservative managements. Two patients already had arm lymphedema after breast-conserving surgery and adjuvant chemotherapy, and the symptom of arm lymphedema was persistent during breast irradiation and follow-up. Two patients had grade 2 pneumonitis one month after breast irradiation and were cured with steroid treatment.

**Table 4:** **Acute treatment toxicity of breast irradiation using TomoDirect**

**Complication**	**Grade**		
	**2**	**3**	**4**
	Number (percent) of patients		
Hematologic			
Leucopenia	0	0	0
Anemia	0	0	0
Thrombocyopenia	0	0	0
Non hematologic			
Dermatitis	41 (26.5)	6 (3.9)	0
Radiation pneumonitis	2 (1.3)	0	0
Pericarditis	0	0	0
Arm lymphedema	2 (1.3)	0	0

## Discussion

Helical TomoTherapy, which enabled simultaneous IMRT and IGRT, has achieved encouraging clinical outcomes in terms of tumor response, survival, and toxicity in head and neck and prostate cancer [9,10]. However, it is not easy to apply continuously-rotating TomoTherapy to breast cancer patients, since it is accompanied by low-dose irradiation on both lungs and can result in radiation pneumonitis [11].

TomoDirect, which uses static gantry positions, combined with simultaneous couch translation and multi-leaf collimator modulation makes it possible to irradiate the whole breast without low-dose radiation to lungs in breast cancer patients. The static gantry angles of TomoDirect are identical to the tangential beam angles of conventional linear accelerator. The difference between TomoDirect and conventional 3-dimensional conformal radiotherapy (3DCRT) is that the patient is stationary during breast irradiation using the conventional linear accelerator and the patient slides through the intensity-modulated beam during breast irradiation using TomoDirect. Reynders *et al.* assessed the dosimetric parameters of TomoDirect and 3DCRT. Compared with conventional radiotherapy, TomoDirect provided an adequate PTV coverage with a significant reduction of high-dose radiation to the OAR [5]. In our prospective study, TomoDirect achieved perfect target dose coverage in all patients except one patient with a large breast: ≥98% of the PTV received ≥95% of the prescribed dose and ≤5% of the PTV received ≥110% of the prescribed dose. None received <115% of the prescribed dose to the PTV. The conformality of TomoDirect irradiation was good with an average RCI value of 1.01 and the homogeneity of TomoDirect irradiation was favorable with an average rDHI value of 0.89. The TomoDirect option enables specified beam angles, reducing planning time of IMRT compared with helical TomoTherapy. Moreover, the beam-on time of TomoDirect during breast irradiation is less than helical TomoTherapy and comparable to that of 3DCRT [6]. In our study, median beam-on time was just 175 seconds. These considerations could make TomoDirect a useful option in the radiation oncology department which only equipped with helical TomoTherapy without conventional linear accelerator.

The radiation dose delivered to the organs at risk, such as the ipsilateral lung, heart, and contralateral breast, was evaluated by dosimetric values of mean dose and V_10-50Gy_. The incidence of moderate to severe radiation pneumonitis after radiotherapy ranges from 0% to 37% [12]. This wide variation reflects the different types of radiotherapy and the presence or absence of neoadjuvant or adjuvant chemotherapy, which may influence the risk of radiation pneumonitis. The risk of radiation pneumonitis seems to increase as the cumulative dose of radiation to the normal lung tissue increases, and the mean lung dose and V_20Gy_ are considered reliable dosimetric predictors for the occurrence of radiation pneumonitis [13]. A mean lung dose <10 Gy and V_20Gy_ <20% is associated with the radiation pneumonitis probability of <3% [14]. In our trial, dosimetric values for the ipsilateral lung were mean dose of 4.72 Gy, V_20Gy_ of 7.23%, and V_30Gy_ of 5.33%, and only two patients (1.3%) had grade 2 radiation pneumonitis one month after breast irradiation using TomoDirect.

Whole breast irradiation after breast-conserving surgery is sometimes accompanied by radiation-induced heart injury. Especially, the pericardium near the tangential photon beam is a vulnerable area for radiation damage. The dose-volume relationship and mean dose for the heart is significantly associated with radiation-induced heart disease [15]. According to the quantitative analysis of normal tissue effect in the clinic (QUANTEC) review, V_25_ < 10% correlates with a long-term cardiac mortality <1%, an overly safe risk estimate based on model predictions [13]. The dose–response relationship for pericarditis was suggested by the QUANTEC review, and predicted that mean dose <26 Gy and V_30_ < 46% corresponded to a pericarditis probability of <15%. In our study, mean radiation dose for the heart was just 1.09 Gy and V_30_ and V_20_ values were <1%. Thus, in our trial no patient had radiation-induced heart disease during breast irradiation and 3 months after the end of breast irradiation.

The occurrence of contralateral breast carcinoma after breast irradiation has been extensively investigated. Scattered radiation beams certainly reached the opposite breast, and this could bring about theoretical carcinogenesis on the contralateral breast. However, there was no distinct evidence that scattered radiation during breast irradiation was significantly associated with the occurrence of contralateral breast cancer. In a case–control study including a cohort of 41,109 women diagnosed with breast cancer, the risk of a second cancer in the opposite breast was significantly increased among women who underwent irradiation at a relatively young age (<45 years) [16]. However, Fisher *et al.* reported that the risk for an event of contralateral breast cancer among a group treated with lumpectomy and irradiation, as compared with the total-mastectomy group, was not significantly different in the 20-year follow-up of a randomized trial [2]. In another study, there was no statistically significant difference of the 20-year crude cumulative incidence for contralateral breast cancer between breast-conserving surgery and radiation versus mastectomy groups [3]. In our trial, TomoDirect delivered a mean dose of 0.19 Gy to the contralateral breast and _V10–50_ values were all <0.5%. Thus, this scattered radiation to the contralateral breast might not be carcinogenic [17,18].

Although our trial showed very favorable results for target coverage and normal tissue sparing with TomoDirect in early breast cancer patients, we acknowledge that our trial had a number of limitations. First, our study should be understood in view of the inherent biases of a short-term follow-up design. We did not evaluate chronic adverse effects. Second, we did not evaluate the recurrence and survival outcome due to the short-term follow up. Thus, we do not suggest that TomoDirect using SIB in early breast cancer is as effective as conventional breast irradiation in terms of survival and recurrence. For the clear-cut assessment of long-term complication such as the occurrence of contralateral breast cancer, recurrence, and survival, a follow-up time exceeding 10 years is required.

SIB technique using TomoDirect in early breast cancer patients showed acceptable toxicity profiles and optimal results in terms of target coverage and normal tissue sparing. Survival, tumor recurrence, and concerns for the risk of contralateral breast carcinoma due to scattered radiations during breast irradiation must be evaluated through a long-term follow-up study.
